# Reply to: Letter to the Editor regarding "Covid-19 transmission in fitness centers in Norway—a randomized trial"

**DOI:** 10.1186/s12889-022-14799-x

**Published:** 2022-12-27

**Authors:** Lise M. Helsingen, Magnus Løberg, Michael Bretthauer, Mette Kalager

**Affiliations:** Clinical Effectiveness Research Group, Institute of Health and Society, University of Oslo, Oslo, Norway

**Keywords:** Covid-19, Randomized trial, Public health

## Abstract

In this correspondence we respond to critique of our randomized trial of Covid-19 transmission in fitness centers. The trial was performed in Norway during May and June 2020.

We appreciate the interest that Valberg and co-workers shows in our randomized trial about training in fitness centers during the Covid-19 pandemic and welcome a discussion about design and interpretation [1, 2].

We agree with biostatisticians Morten Valberg and co-workers that the incidence of Covid-19 was low during the study. Obviously, we cannot influence incidence of disease in the future when we plan trials. We believe this inability is common for researchers who plan and conduct prospective trials which play out after they are planned.

The rationale for our choices of intervention type and duration has been explained in detail in the trial protocol which is available to all readers on request, including Valberg and co-workers. Contrary to what Valberg and co-workers claim, we have also addressed the challenges related to low incidence of disease and timing and content of intervention in the Discussion of the paper so that all readers are aware of them. Please see Discussion: “Our trial was limited by the low number of events in both arms.” and “It is, however, unclear if our findings would apply to areas with higher SARS-CoV-2 and Covid-19 incidence rates.”

The incidence of Covid-19 during the intervention period of the trial was high enough for the Norwegian government to require closure of fitness centers in the trial areas. Therefore, regardless of whether one considers the actual incidence as “low” or “high”, it was certainly high enough to restrict access by emergency laws, which we believe is certainly sufficient to warrant a trial like ours.

Valberg and co-workers suggest we could have performed simulation studies to guide the design and sample size calculation for the trial. We agree that this may help guide uncertainties arising with a novel virus. However, we are uncertain if it would solve the issues inherently related to prospective trials, due to the uncertainty of modelling studies in situations like the one we were facing, with a largely unknown virus, little data on spread and incidence development, and no prior data on how training center access would influence Covid-19 incidence. As known, the value of simulations models are highly dependent on several assumptions.

We agree with Valberg and co-workers that it is challenging to perform randomized trials in areas such as infectious diseases and interventions such as access to specific venues, where blinding cannot be performed and exposure may occur both within and across treatment groups. Our trial has these challenges in common with every trial in this area and with this intervention type, and limitations arising from this are duly noted in the Discussion: “The trial was not designed to establish any protective effect of training against Covid-19 and these results should be interpreted with caution. A possible explanation for the difference may be alternative exercise patterns in uncontrolled environments in the individuals in the no-training arm who did not have access to a fitness center. This deserves further study.”

Valberg and co-workers are concerned that our conclusion based on the ITT analyses might be wrong. Our drop-out and non-compliance rates were low, as we have reported in the paper. We acknowledge that drop-out and non-compliance might be particularly challenging to handle in non-inferiority trials where these mechanisms typically bias the result towards the non-inferiority conclusion. We did, however, perform sensitivity analyses to explore how many more positive tests would be needed to arrive at another conclusion: “14 participants in the training arm and none in the no-training arm had to test positive before the upper bound of the 95% confidence interval crossed the predefined non-inferiority margin of 1% (risk difference 0.69, 95% confidence interval 0.32 to 1.06%)”. Further, as all relevant clinical endpoints were gathered through registries, drop-out was zero for these important endpoints. We have pointed this out in the Discussion: “Compliance with SARS-CoV-2 RNA testing was higher in the training arm (89%) than in the no-training arm (71%). However, disease endpoints in the trial were gathered through complete hospital registries and are not prone to self-reporting bias. Also, the number of individuals who withdrew consent after randomization was small (18 in the training arm and 43 in the no-training arm). Finally, sensitivity analyses investigating effect of missing data confirm the robustness of the estimates.”

Valberg and co-workers are concerned with generalizability to other disease prevalence/incidence, other areas of the World, seasons of the year etc. We agree, and we have clarified that already in the Discussion: “The rate of new positive tests outside the trial in Oslo during the trial period was not substantially different to that of many states and counties in the United States reported in the same period (e.g. positive test rate per 100,000 individuals in the week of June 15 to 21, 2020 was 13 in Maine, 25 in New Jersey, and 22.5 in Massachusetts) [13]. It is, however, unclear if our findings would apply to areas with higher SARS-CoV-2 and Covid-19 incidence rates.”

In conclusion, while we appreciate Valberg and co-worker’s arguments, we believe that we have addressed the raised issues appropriately in the paper, we have flagged challenges and issues of generalizability, and we have asked for caution in interpretation of our results. We agree that randomized testing during an ongoing epidemic is not trivial, which is why we conducted the current study in fitness centers in Norway. We believe it is even more trivial not to study non-pharmaceutical interventions, which have been implemented worldwide without any requirement to show scientific evidence for benefits and harms.

On behalf of all the study authors, 

Lise M. Helsingen, Magnus Løberg, Michael Bretthauer and Mette Kalager

## Data Availability

Not applicable.
